# Risk Factors for Neonatal Sepsis in Public Hospitals of Mekelle City, North Ethiopia, 2015: Unmatched Case Control Study

**DOI:** 10.1371/journal.pone.0154798

**Published:** 2016-05-10

**Authors:** Destaalem Gebremedhin, Haftu Berhe, Kahsu Gebrekirstos

**Affiliations:** 1 Dr. Tewelde Legesse Health Sciences College, Mekelle, Ethiopia; 2 Mekelle University, College of Health Sciences, Mekelle, Ethiopia; University of Southern California, UNITED STATES

## Abstract

**Objectives:**

Neonatal sepsis is a leading cause of neonatal morbidity and mortality, particularly in the developing countries. Delays in the identification and treatment of neonatal sepsis are among the main contributors to the high mortality. The aim of this study was to determine the risk factors of neonatal sepsis in public hospitals of Mekelle City, Tigray Region, North Ethiopia, 2015.

**Methods:**

A hospital based case control study was done in public hospitals of Mekelle City, Tigray region. Cases were neonates who had sepsis with their index mothers and controls were neonates who hadn’t had sepsis with their index mothers. Hematologic findings were used to diagnose sepsis once the neonates were being clinically suspected. Cases and controls were selected using the systematic sampling technique. Data were entered using Epi info version 7 and then analyzed using SPSS window 20. The binary logistic regression model was used to test the association between dependent and independent variables and multivariable logistic regression was used to identify the associated risk factors to neonatal sepsis.

**Findings:**

A total of 78 cases and 156 controls were included in this study. More than three quarters (76.8%) of cases had early onset sepsis. The multivariable logistic regression analysis showed that the possible risk factors of neonatal sepsis in this study were; history of maternal urinary tract infection or sexually transmitted infection [AOR = 5. 23; 95% CI (1.82, 15.04)], prolonged rupture of membrane [AOR = 7. 43; 95% CI (2.04, 27.1)], Place of delivery; health center delivery [AOR = 5. 7; 95% CI (1.71, 19.03)], intrapartum fever [AOR = 6. 1 95% CI (1.29, 28.31)], APGAR score <7 at 5^th^ minute [AOR = 68. 9; 95% CI (3.63, 1308)] and not crying immediately at birth [AOR = 124. 0; 95% CI (6.5, 2379)].

**Conclusion:**

Both maternal and neonatal factors had contributed to the risk of neonatal sepsis. Strengthening of the existing risk based prevention strategies as well as improvement of institutional delivery practices are crucial.

## Introduction

According to the international pediatric sepsis consensus conference of 2005, neonatal sepsis is defined as systemic inflammatory response syndrome in the presence of or as a result of suspected or proven infection in a neonate. Infection could be of bacterial, viral, fungal, or rickettsial origin [1]. Neonatal sepsis encompasses various systemic infections of the newborn, such as septicemia, meningitis, pneumonia, arthritis, osteomyelitis etc.[2].

Depending on the onset age of the disease, neonatal sepsis is divided into early neonatal sepsis or late onset sepsis. Early neonatal sepsis (EOS) is mainly due to organisms acquired before and during delivery (or maternal fetal infection), where as late onset sepsis (LOS) is due to organisms acquired after delivery from the environment (nosocomial or community sources). However, there is little consensus as to what age limits apply, with early onset ranging from 48 hours to 7 days after delivery [3].

In the last two decades, a remarkable progress has been shown on maternal and child deaths, but the neonatal health is a part of the 'unfinished agenda'. The world is experiencing an increase in the proportion of under five death occurring in the neonatal period. Yet despite the neonatal deaths are preventable, they are concentrated in the world's poorest countries. And 85% of all the neonatal were occurred in low and middle income countries (LMICs) even though they are home to only 62% of the world's newborns [4, 5].

Globally 15% of neonatal deaths are caused by neonatal sepsis and particularly it is a major concern in the LMICs. Furthermore, it is also associated with increased medical costs, prolonged hospital stay and potentially poor long-term neurodevelopmental outcomes. Surviving infants, approximately one-fourth of neonates, have significant neurological sequelae as a consequence of CNS involvement, septic shock or hypoxemia secondary to severe parenchymal lung disease despite prompt instigation of effective antibiotic therapy. Despite of this, the world is witnessing a steady decline in the number of neonatal deaths due to sepsis, the neonatal mortality from sepsis declined by only 28 percent [4–6].

On the continent of Africa, Seventeen percent of neonatal deaths in sub Saharan Africa are attributed to neonatal sepsis as compared to only 6% of neonatal deaths are due to sepsis in high income countries. Neonatal sepsis, the major newborn killer in Ethiopia, accounts for more than one third of neonatal deaths. In Tigray region, neonatal sepsis is also a major cause of neonatal morbidity and deaths next to prematurity and birth asphyxia. It causes 24% of neonatal deaths with an incidence rate as high as 10% per 1000 live births [5, 7–9].

Many factors contribute to the high mortality due to infections due to delays in the identification and treatment of newborns with infection, specifically; including under-recognition of illness, delay in care seeking at the household level, delay in initiation of treatment, and lack of access to both appropriately trained health workers and to high quality services to manage sepsis. It is particularly poignant that many neonatal deaths occur in the community, without the newborn ever having contact with the appropriate health services [10, 11].

Indeed, strategies that can prevent and treat neonates with sepsis are essential to accelerate the progress of newborn survival. In many developing country settings, however, the identification and treatment of newborns with infection is unsatisfactory and epidemiological data from developing countries showed differences in the incidence, risk factors, pattern and antimicrobial sensitivities of pathogens and mortality from that of developed countries. Identification of risk factors and early institution of therapy, thereby can improve neonatal mortality and morbidity [5, 12].

Many centers have studied the common causative agents of neonatal sepsis with their sensitivity patterns. But there are limited studies tried to verify the risk factors of neonatal sepsis in the study area as well as in the country as a whole. Hence, there is a need to carry out a research to come up with the risk factors of neonatal sepsis.

This study, therefore, was aimed to determine the risk factors of neonatal sepsis in public health hospitals in Mekelle City, North Ethiopia.

## Methods

This hospital based unmatched case control study was conducted in Mekelle City, North Ethiopia, which is located at 783km from Addis Ababa (the capital city of Ethiopia) since December 2014 to June 2015. The study was carried out among neonates who were admitted to public hospitals of the city.

The hematological criteria along with the established IMNCI (Integrated Management of Neonatal and Childhood Illness) clinical features of neonatal sepsis were used to diagnose neonatal sepsis in this study. Neonates in the presence of one or more of the established IMNCI clinical features [either of fever (≥37.5°C) or hypothermia (≤ 35.5°C), fast breathing (≥60 breath per minute), severe chest indrawing, not feeding well, movement only when stimulated, convulsion, lethargic or unconscious] along with ≥ 2 of the hematological criteria; total leukocyte count (<4000 or >12000 cells/m^3^, absolute neutrophil count (<1500 cells/mm3 or >7500 cells/mm^3^), erythrocyte sedimentation rate (ESR) (>15/1 h) and platelet count (<150 or >440 cells/m^3^) and who were admitted to pediatric ward or neonatal ICU of the public hospitals of Mekelle City, North Ethiopia during the study period were included with their index mothers as cases. Neonates who were not fulfilled the criteria of sepsis and who were admitted to pediatric ward or neonatal ICU of the public hospitals in Mekelle City, North Ethiopia during the study period were also included with their index mothers as controls.

A two population proportion formula (using open Epi version 2.3.1) was used to estimate the sample size required for the study by considering that the proportion of mothers with UTI/ STI among the controls of 13% (main exposure variable), which was estimated from another study [13], 95% CI, 80% power of the study control to case ratio of 2:1 to detect an odds ratio of 2.87 which was estimated from a study done by others [13]. Accordingly, by adding 5% for the non response rate, 78 cases and 156 controls (a total sample size of 234) was the estimated sample size in this study. Cases and controls were selected using proportional systematic random sampling.

Data was collected using semi structured questionnaire and checklist prepared in English and then translated to the local language, Tigrigna (S1 Appendix). The tool was pretested on 5% (4 cases and 8 controls) of the sample size before the actual data collection period. The data were collected by 4 nurses with previous experiences and after being trained by the principal investigator about the purpose of the study and how to interview as well as fill the questionnaire and checklist properly. Finally, the data collectors collected the data through interviewing the mothers and reviewing neonates’ medical records throughout the data collection period.

The data were checked for completeness, inconsistencies, then entered using Epi-Info version 7 and cleaned and analyzed in SPSS version 20. Cross tabulation was done to see the distribution of cases and controls. The binary logistic regression model was used to test the association between dependent and independent variables. All variables with P value <0.29 in bivariate analysis were included in the multivariable analysis. Magnitude of association was measured by using an odds ratio at 95% confidence interval. Statistical significance was declared at P<0.05. Finally, the data are presented with texts and tables.

### Ethical Consideration

The study was conducted after getting ethical clearance from Mekelle University, College of Health Science Ethical Review Board. Support letter was obtained from Mekelle University to Tigray Regional Health Bureau and from Tigray regional health bureau for respective health institutions. In addition, written informed consent was obtained from study participants to confirm willingness once the consent procedure was approved by the ERB and they were notified that they have the right to refuse or terminate at any point of the interview.

## Results

### Socio-demographic characteristics of respondents

In this study, a total of 78 neonates who had sepsis (cases) with their index mothers and 156 neonates who had no sepsis (controls) with their index mothers were included making a response rate of 100%. The mean (±SD) age of mothers was 26.38 ±5. 52 years range from 18 to 40 years. Twenty one (26.9%) of cases and 28 (17.9%) controls were living in rural areas. Regarding to marital status, 68 (87.2%) cases and 142 (91.0%) of controls were married. Forty eight (61.5%) of cases and 92 (59.0%) of controls were house wives by occupation and 29 (37.2%) cases and 37 (23.7%) of controls had not attended formal education. Concerning to neonates’ socio demographic characteristics, 60 (76.8%) of the cases and 149 (95.5%) controls were found under the age of 7 days. The proportion of male neonates were higher in the cases 56 (71.8%) than controls 86 (55.1%) (**Table 1**).

**Table 1:** Socio demographic characteristics of cases and controls attending public hospitals of Mekelle city, North Ethiopia, 2015.

Variables	Category	Cases n = 78 (%)	Control n = 156 (%)	Total n = 234 (%)
Maternal age	15–20	24(30.8)	24(15.4)	48(20.5)
	21–34	44(56.4)	111(71.2)	155(66.2)
	≥35	10(12.8)	21(13.5)	31(13.2)
Marital status	Single	4(5.1)	5(3.2)	9(3.8)
	Married	68(87.2)	142(91.0)	210 (89.7)
	Divorced/ separated	6(7.7)	9(5.8)	15(6.4)
Religion	Orthodox	67 (85.9)	134(85.9)	201(85.9)
	Muslim	3(3.8)	6(3.8)	9(3.8)
	Protestant	8(10.3)	11(7.1)	19(8.1)
	Catholic	0(0.0)	5(3.2)	5(2.1)
Residence	Urban	57(73.1)	128(82.1)	185(79.1)
	Rural	21(26.9)	28(17.9)	49(20.9)
Ethnicity	Tigray	75(96.2)	152(97.4)	227(97.0)
	Amhara	1(1.3)	2(1.3)	3(1.3)
	Afar	2(2.6)	2(1.3)	4(1.7)
Educational level	No formal education	29(37.2)	37(23.7)	66(28.2)
	Primary school	18(23.1)	47(30.1)	65(27.8)
	Secondary school	18(23.1)	35(22.4)	53(22.6)
	College and higher	13(16.7)	37(23.7)	50(21.4)
Occupation	Housewife	48(61.5)	92(59.0)	140(59.8)
	Civil servant	8(10.3)	29(18.9)	37(15.8)
	Business women	6(7.7)	8(5.1)	14(6.0)
	Others*	16(20.5)	27(17.3)	43(18.3)
Neonate age in days	<7	60(76.9)	149(95.5)	209(89.3)
	7–28	18(23.1)	7(4.5)	25(10.7)
Neonate sex	Male	56(71.8)	86(55.1)	142(60.7)
	Female	22(28.2)	70(44.9)	92(39.3)

### Pregnancy and obstetric history of the study subjects

The median (±IQ range) of parity for both cases and controls was 2±2 ranges from 1 to 12 live births. The majority of the respondents, 154 (98.7%) controls and 76 (97.4%) cases had received antenatal care (ANC) service at least once during the index pregnancy. More than two thirds, 102 (65.4%) of controls and 48 (61.5%) cases were delivered by spontaneous vaginal delivery. This study revealed that the proportion of mothers who had a history of urinary tract infections or sexually transmitted infections (UTI/STI) during the index pregnancy was higher in the cases 40 (51.3%) than controls 21 (13.5%). Similarly, the proportion of mothers who gave birth after 18 hours of rupture of the membrane (PROM) was higher in the cases 24 (30.8%) than controls 6 (3.8%).

### Neonate characteristics

One hundred one (64.7%) controls and 45 (57.7%) cases were delivered at term with normal birth weight. The proportion of neonates who had APGAR (Appearance Pulse Rate Grimacy Activity Respiration) score < 7 at first minute was higher in the cases 31 (39.7%) than controls 12 (7.7%). Similarly, 35 (44.9%) of cases and 13 (8.3%) of controls were resuscitated at birth.

### Maternal and neonatal risk factors of neonatal sepsis

After applying both bivariate and multivariable logistic regression, only six variables had shown an overall significant effect on risk of neonatal sepsis at the 5% level of significance (**Table 2**).

**Table 2:** Bivariate and multivariable logistic regression analysis result of the study.

Variables	Category	Cases n = 78(%)	Control n = 156(%)	COR [95%CI]	AOR [95%CI]
Maternal age	21–34	44(56.4)	111(71.2)	1.0	1.0
	18–20	24(30.8)	24(15.4)	2.52[1.30,4.91]*	1.79[0.63, 5.05]
	≥35	10(12.8)	21(13.5)	1.20[0.52, 2.76]	0.51[0.11, 2.34]
Residence	Urban	57(73.1)	128(82.1)	1.0	1.0
	Rural	21(26.9)	28(17.9)	1.68[0.88, 3.21]	0.83[0.27, 2.54]
Educational level	Illiterate	29(37.2)	37(23.7)	1.90[1.10,3.43]*	1.83[0.60, 5.60]
	Literate	49(62.8)	119(76.3)	1.0	1.0
Place of delivery	Home	1(1.3)	2(1.3)	1.26[0.1, 14.16]	6.28[0.35, 113.0]
	Health centers	21(26.9)	13(8.3)	4.07[1.9, 8.68]*	5.7[1.71, 19.0]**
	Hospital	56(71.8)	141(90.4)	1.0	1.0
Mode of delivery	SVD	48(61.5)	102(65.4)	1.0	1.0
	Instrumental	9(11.5)	6(3.8)	3.19[1.1, 9.47]*	3.39[0.54, 21.13]
	CS	21(26.9)	48(30.8)	0.93[0.5, 1.72]	0.99[0.36, 2.72]
Hypertensive disorder	Yes	10(12.8)	3(1.9)	7.5[2.0, 28.12]	4.24[0.53, 33.8]
	No	68(87.2)	153(98.1)	1.0	1.0
Bleeding disorder	Yes	8(10.3)	4(2.6)	4.34[1.3, 14.9]*	2.1[0.34, 13.0]
	No	70(89.7)	152(97.4)	1.0	1.0
UTI/STI	Yes	40(51.3)	21(13.5)	6.8[3.6, 12.82]*	5.23[1.8, 15.04]**
	No	38(48.7)	135(86.5)	1.0	1.0
PROM	Yes	24(30.8)	6(3.8)	11.1[4.3, 28.7]*	7.43[2.04 27.1]**
	No	54(69.2)	150(96.2)	1.0	1.0
Pervaginal examination	≤3	53(67.9)	127(81.4)	1.0	1.0
	>3	25(32.1)	29(18.6)	2.07[1.1, 3.86]*	1.22[0.40, 4.91]
Intrapartum fever	Yes	22(28.2)	7(4.5)	8.36[3.4, 20.7]*	6.05[1.29, 28.3]**
	No	56(71.8)	149(95.5)	1.0	1.0
Foul smelling liquor	Yes	7(9.0)	5(3.2)	2.98[0.92, 9.7]	0.44[0.04, 4.91]
	No	71(91.0)	151(96.8)	1.0	1.0
Neonate sex	Male	56(71.8)	86(55.1)	2.07[1.2, 3.72]*	1.5[0.6, 3.60]
	Female	22(28.2)	70(44.9)	1.0	1.0
Gestational age in weeks	37–42	52(66.7)	122(78.2)	1.0	1.0
	<37	22(28.2)	27(17.3)	1.91[0.99, 3.66]	1.54[0.44, 5.45]
	>42	4(5.1)	7(4.5)	1.34[0.38, 4.78]	7.14[1.32, 38.6]
Birth weight in gram	1500 to 2500	24(30.8)	42(26.9)	1.21[0.66, 2.19]	1.15[0.36, 3.73]
	≥2500	54(69.2)	114(73.1)	1.0	1.0
APGAR score in the first minute	<7	31(39.7)	12(7.7)	7.9[3.76, 16.64]	0.33[0.04, 3.07]
	≥7	47(60.3)	144(92.3)	1.0	1.0
APGAR score in the fifth minute	<7	17(21.8)	3(1.9)	14.2[4.0, 50.3]*	68.9[3.6,1307.9]**
	≥7	61(78.2)	153(98.1)	1.0	1.0
Cries immediately after birth	Yes	53(67.9)	153(98.1)	0.04[0.01,0.14]*	1.0
	No	25(32.1)	3(1.9)	1.0	124.0[6.5,2379] **
Resuscitated at birth	Yes	35(44.9)	13(8.3)	8.95[4.4, 18.4]*	0.47[0.06, 3.60]
	No	43(55.1)	143(91.7)	1.0	1.0

Accordingly, place of delivery showed significant association with the risk of onset of neonatal sepsis. The odds of having neonates with sepsis among mothers who gave birth at health center was 5.7 times higher compared to those who gave birth in hospitals [AOR = 5. 70; 95% CI (1.71, 19.00)].

History of UTI/STI during the index pregnancy also showed a statistical significant association with neonatal sepsis. This study showed that, neonates born to mothers who had UTI/STI during the index pregnancy had 5 times higher odds of developing sepsis compared to those neonates born to mothers who did not have a UTI / STI during the index pregnancy [AOR = 5. 23; 95% CI (1.82, 15.04)].

Prolonged rupture of membrane (PROM) and intrapartum fever had significant association with risk of neonatal sepsis. The odds of neonatal sepsis among mothers who gave birth after 18 hours of rupture of membrane was 7.4 times higher than those mothers who gave birth before 18 hours of rupture of membrane [AOR = 7.43; 95% CI (2.04, 27.71)]. Similarly, those neonates who were born to mothers who had fever during labor had 6 times higher odds developing sepsis compared to those neonates whose mothers did not have intrapartum fever [AOR = 6. 08; 95% CI (1.29, 28.31)].

APGAR score at 5^th^ minute and immediate cry after birth were also significantly associated with risk of neonatal sepsis in the multivariable analysis. Neonates who had APGAR score <7 at 5^th^ minute had higher odds of developing sepsis compared to neonates who had APGAR score ≥7 [AOR = 68. 9; 95% CI (3.63, 1307.90]. Similarly, neonates who cried immediately at birth were 99% less likely to develop sepsis as compared to neonates who did not cry immediately [AOR = 0. 01; 95% CI (0.00, 0.16)].

## Discussion

This study was aimed to assess maternal and neonatal risk factors of neonatal sepsis in order to contribute to tackle the burden of the disease and its associated problems. This study has attempted to look the determinants of neonatal sepsis by incorporating as many risk factors as possible. In this study, three fourth of the cases (76.9%) were with early onset neonatal sepsis (<7 days) which is more or less comparable with the studies conducted earlier in our country in Gonder (2012) and Bishoftu (2014) which was 81.8% and 81.4% respectively [14, 15].

The finding of this study showed that both maternal and neonatal factor had a significant effect on the risk of neonatal sepsis, though all factors did not show similar effects as findings of the previous studies.

This study showed that a significant number of neonates born at health center developed sepsis with 5.7 times higher odds of developing sepsis compared to neonates born in hospitals. A previous study conducted in Bishoftu, Oromia (2014) also suggested that the proportion of neonates who were born at health center had higher risk compared to home delivery [15]. This might be due to the reason that neonates who were delivered at health center had less likely to be screened based on a risk approach based and to be treated with intrapartum antibiotic prophylaxis [10].

In this study, more than half (51.3%) of the cases were born to mothers who had a history of UTI/STI during the index pregnancy with five times higher odds of developing sepsis compared to neonates born to mothers who did not have a UTI / STI diagnosis. This finding is more or less comparable with the findings of studies conducted previously in India (2005) (OR = 14. 3 [95%CI 1.97, 79.24]), Ghana (2014) (OR = 3. 01 [95% CI 1.48, 6.43]) and Bishoftu, Ethiopia (2014) which revealed that maternal UTI/ STI (OR = 12. 9; 95% CI [1.49, 5.5]) was a significant factor for the development of neonatal sepsis [13, 15, 16]. This study indicated that 82.5% of those neonates who were born to mothers who had UTI/STI and developed sepsis are found within the age range below 7 days. This finding may support for the reason that maternal UTI/STI is often associated with early onset neonatal sepsis, especially if untreated during the third trimester pregnancy or labor, and it may be associated with neonatal sepsis following the colonization of the birth canal by the infectious agent [15, 17].

Prolonged rupture of membrane (≥ 18 hours) had shown a significant effect on risk of neonatal sepsis with the likelihood of sepsis was 7.4 times higher among neonates born to mothers who had PROM ≥ 18 hours compared to those neonates born before 18 hours of rupture of membrane. One third (30.8%) of cases were born to those mothers who had PROM. Similar findings were also observed in earlier studies conducted in different parts of the world [14, 17–22]. Early rupture and prolonged labor increases the chance of ascending microorganisms from the birth canal into the amniotic sac and fetal compromise as well as asphyxia which frequently leads to sepsis [13, 17].

Intrapartum fever had shown a significant effect on the development of neonatal sepsis in the present study. Neonates who were born to mothers who had fever during labor had 6 times higher odds of developing sepsis compared to neonates born to mothers who did not have fever during labor. This is consistent with the study conducted previously in Pakistan (2014) and Bangladesh (2011) which revealed intrapartum fever was an independent predictor of neonatal sepsis [17, 21]. Intrapartum fever is indicative of maternal infections that are frequently transmitted to the baby in utero or during passage through the canal which usually causes early onset sepsis [17,23].

This study showed that APGAR score at 5^th^ minute had a strong significant effect on the development of neonatal sepsis. Similar observations are also shown in the studies conducted in India (1997), Saudi Arabia (1997), Bangladesh (2011) and Washington (1985) which indicated that APGAR score at 5^th^ minute had a strong effect on risk of neonatal sepsis [17, 18, 23, 24]. Asphyxia causes an immunological insult and resuscitation procedures following birth asphyxia tend to explore newborns to pathogenic microbes [17, 18].

Immediate crying at birth currently showed a significant association with neonatal sepsis. The present study indicated that neonates cried at birth had 99% less likely to be suffering from sepsis compared to those who did not cry at birth. This finding is in line with the previous finding, reported in Ghana which showed that neonates cried at birth had 92% less likely to develop sepsis than neonates who did not cry at birth [13]. This difference might be due to the nature of crying as the physiologic events and changes associated with it.

Prematurity and low birth weight are the well established neonatal risk factors in industrialized and developing countries[13,16, 18–21,23, 25–28]. Unfortunately, this study did not observe an association between preterm or low birth weight and risk of neonatal sepsis. Few other studies, conducted in Bangladesh (2011) and Bishoftu (2014) also observed that low birth weight had an insignificant effect on the risk of neonatal sepsis [15, 17]. Smaller sample size might influence the result along with health service related factors and study design.

Residence, parity, ANC service utilization, mode of delivery and foul smelling liquor were not found to be predictors of neonatal sepsis in this study. This is in contrary to the findings of studies on risk factors of neonatal sepsis in different parts of the world indicated that these factors had an influence on neonatal sepsis [13–25]. The reasons for these differences may range from the background of the participants, access to health facilities and health professionals.

### Limitation of the study

- Since the study is done on admitted neonates, thus results might lack generalizabilility to the entire population in the catchment area.
- Lack of microbiology, diagnosis by different caregivers could arise errors in the identification of cases and controls in the study.

## Conclusion

In conclusion, this study has found that both maternal and neonatal factors had contributed to the risk of neonatal sepsis. History of maternal UTI/STI, place of delivery, PROM, intrapartum fever, low APGAR score at 5^th^ minute and not crying immediately at birth were identified as possible independent risk factors of neonatal sepsis. On the other hand; Residence, parity, ANC service utilization, mode of delivery, foul smelling liquor, prematurity and low birth weight were not found to be statistically associated with risk of neonatal sepsis. This study has also observed that the onset of neonatal sepsis was higher in the first week of neonate’s life. Strengthening of antenatal screening of mothers, perinatal care of newborns and interventions of babies born with complications are recommended.

## Supporting Information

S1 Appendix Study Questionnaire in English and Tigrigna. (DOCX)

S1 Dataset Questionnaire Results Data in SPSS Format. (SAV)
