# Supplementary material for: Risk Factors for Neonatal Sepsis in Public Hospitals of Mekelle City, North Ethiopia, 2015: Unmatched Case Control Study
Source: PLoS One. 2016 May 10;11(5):e0154798. doi: 10.1371/journal.pone.0154798 (PMC4862626; doi:10.1371/journal.pone.0154798)
Supplement: S1 Appendix — (DOCX) [file pone.0154798.s001.docx]

## I: Consent form

In signing this document, I am giving my consent to participate in the study titled “ risk factors of neonatal sepsis in public hospitals of Mekelle city, North Ethiopia”.

I have been informed that the purpose of this study is to identify neonatal sepsis risk factors. I have understood that participation in this study is entirely voluntarily. I have been told that my answers to the questions will not be given to anyone else and no reports of this study ever identify me in any way. I have also been informed that my participation or non-participation or my refusal to answer questions will have no effect on me. I understood that participation in this study does not involve risks.

I understood that Mr. Destaalem Gebremedhin is the contact person if I have questions about the study or about my rights as a study participant.

Respondent’s signature_________________________________

Date of interview: _______________ Time started: _______ Time finished: _________

Interviewer Name_________________________Signature___________Date____________

Supervisor’s name ________________ signature ________

Results of interview questionnaire

1. Completed

2. Refused

3. Partially completed

## II: English version Questionnaire

Mekelle University, College of Health Sciences, Department of Nursing

A questionnaire to determine maternal and neonatal risk factors associated with neonatal sepsis among mothers and their index neonate in public hospitals of Mekelle city, North Ethiopia.

1. Questionnaire ID number___________

2. Address: kebele ______________

3. Name of health facility______________

Note: Encircle from the given option and write if any other idea or answer is given

PART I. Socio-demographic characteristics of mothers with their index neonates (age 0-28 days)

| No. | Question | Response | Skip |
| --- | --- | --- | --- |
|  | Mother’s age | _________(in years) |  |
|  | Marital status | 1. Single 2. Married 3. Widow 4. Divorced 5. Separated 6. Cohabitated |  |
|  | What is your religion? | 1. Orthodox  2. Muslim  3. Catholic  4. Protestant  5. Other (specify)__________ |  |
|  | Ethnicity | 1. Tigray 2. Amhara 3. Other (specify) |  |
|  | Residence | 1. Urban 2. Rural |  |
|  | Maternal education | 1. No education  2. Primary  3.Secondary  4. college and higher |  |
|  | Occupation of mother | 1. Housewife  2. Civil servants  3. Business woman  4. Private Organization  5. Daily laborer  6. Student |  |
|  | Monthly income of the household | ________ in Ethiopian Birr |  |
|  | Neonate’s age | __________in days |  |
|  | Neonate’s sex | Male Female |  |
| PART II. Maternal health related factors | | | |
|  | Parity | ______ in number |  |
|  | Did you visit health facility for  ANC during your pregnancy for this neonate? | 1. Yes 2. No | If 'no' skip  to 114 |
|  | If yes, how many times did you receive antenatal care during your time of pregnancy for this neonate | _____ times |  |
|  | Where did you gave birth to this  neonate /Place of delivery | 1. Home  2. Hospital  3. Health center  4. Other (specify)___ | If in 'home', skip to 116 |
|  | If the place of delivery is in hospital  or health center, what was the type of delivery? | 1. Spontaneous Vaginal delivery 2. Instrumental vaginal delivery 3. Caesarean section |  |
|  | Who helped you during delivery? | 1. TBA  2. HEW  3. Health professional  4. Relatives  5. Other (specify)_________ |  |
|  | What was the duration of labor | ____________ in hours |  |
|  | How many times did the birth attendant performs vaginal examination | _________ times |  |
|  | Did you have any fever during the time of this labor | Yes ________ No________  Specify _____ |  |
|  | Did the amniotic fluid was foul smelling | Yes ________ No _____ |  |
|  | Did you have pregnancy related hypertension PIH/ Eclampsia during the pregnancy of this neonate? | Yes ______ No _____  Specify _____ |  |
|  | Did you have any bleeding during the pregnancy of this neonate?/ APH | Yes ______ No _____  Specify _____ |  |
|  | Did you have any UTI/STI during the pregnancy of this neonate? | Yes ______ No _____  Specify _____ |  |
| Thank you for your participation! | | | |

## III: A checklist on neonatal health related factors

|  | Birth order | …………………^th^ |
| --- | --- | --- |
|  | Gestational age | ______ in weeks |
|  | APGAR score | At 1^st^ minute_____  At 5^th^ minute_____ |
|  | Birth Weight at birth | _______ in grams |
|  | Did the neonate cries immediately after birth? | Yes _____ No_____ |
|  | Did the neonate resuscitated at birth? | Yes ______ No ____ |
| IMNCI clinical criteria for diagnosis of neonatal sepsis | | |
|  | Convulsions | Yes ______ No ____ |
|  | Respiratory rate > 60 breaths/min | Yes ______ No ____ |
|  | Severe chest in drawing | Yes ______ No ____ |
|  | Nasal flaring | Yes ______ No ____ |
|  | Grunting | Yes ______ No ____ |
|  | Bulging fontanels | Yes ______ No ____ |
|  | Pus draining from the ear | Yes ______ No ____ |
|  | Redness around umbilicus extending to the skin | Yes ______ No ____ |
|  | Temperature >37.7^o^C or <35.5^o^C | Yes ______ No ____ |
|  | Lethargic or unconscious | Yes ______ No ____ |
|  | Reduced movements | Yes ______ No ____ |
|  | Not able to feed | Yes ______ No ____ |
|  | Not attaching to breast | Yes ______ No ____ |
|  | No sucking at all | Yes ______ No ____ |
| Laboratory investigations findings | | |
|  | Complete blood count (CBC) | 1. Total WBC_________ /mm^3^ 2. ANC _____________/mm^3^ 3. ESR ___________/1hr 4. Platlet count____________cells/m^3^ |
|  | Blood culture | Identified bacteria___________ |
| *WBC=white blood cells, ANC= absolute neutrophil count, ESR= erythrocyte sedimentation rate | | |

## IV: ናይ ስምምዕነት ቅጥዒ ቅድመ መፅናዕቲ ሕቶን መልስን (Tigrigna Version Informed concent)

ኣብ ከተማ መቐለ ኣብ ዝርከባ ሆስፒታላት ረክሲ ሕንጦ ከምፅኡ ዝክእሉ ጉዳያት ኣብ መንጎ ትሕቲ 28 መዓልቲ ዝዕድመኦም ህፃናት ብዝብል ስያሜ ኣብ ዝካየድ መፅናዕቲ ንምስታፍ ዝተሰማማዕኹ ኮይነ፣ ነዞም ዝስዕቡ ዋኒናት ኣብ ግምት ብምእታው እዩ፡፡ ዕላማ ናይዚ መፅናዕቲ ኣብ ከተማ መቐለ ኣብ ዝርከባ ሆስፒታላት ረክሲ ሕንጦ ከምፅኡ ዝክእሉ ጉዳያት ንምፍላይ ምኻኑ ብምርዳእ፣ እዚ ቃለ መሕትት ብድሌት ጥራሕ ዝግበርን ምሽጥሩ ዝተሓለወን ምኻኑ ብምእማን ከምኡ ውን ምስታፈይ፣ ዘይምስታፈይ ኣባይ ምንም ዓይነት ተፅዕኖ ከምዘይብሉ ኣብ ግምት ብምእታው፣ ኢሉ ውን ኣባይ ዘምፅኦ ሳዕቤን ከምዘየለ ብምርዳእ፣ ኣብ መወዳእታ እውን ነዚ መፅናዕቲ ዝምልከት ሕቶ እንተለኒ ወይ ድማ ናይዚ መፅናዕቲ ውፅኢት ክፈልጥ እንተደልየ ንበዓል ዋና እዚ መፅናዕቲ ኣይተ ደስታኣለም ገብረመድህን ኣብ ላዕሊ ብዝተጠቀሰ ኣድራሻ ምጥያቕ ከምዝክእ ብምእማን ፤ ኣብዚ መፅናዕቲ ንምስታፍ ፍቃደኛ እየ፡፡

ናይ ተሓታታይ ፌርማ…………………………

ዝተሐተተሉ ዕለት-----------------ዝተጀመረሉ ሰዓት-------------ዝተወደአሉ ሰዓት----------------

ናይ ሐታታይ ሽም---------------------------------------------------- ፊርማ-----------------

ሽም ተቆፃፃሪ-------------------- ፊርማ-----------------

ውፅኢት ቃለ መሕትት

1. ዝተማልአ

2. ዝተነፀገ

3. ዘይተማለአ

## V: ትግርኛ መሕትት(Tigrigna version Questionaire)

መቐለ ዩኒቨርሲቲ ኮሌጅ ጥዕና ሳይንስ ክፍሊ ትምህርቲ ነርሲንግ

ኣብ ከተማ መቐለ ኣብ ዝርከባ ሆስፒታላት ረክሲ ሕንጦ ከምፅኡ ዝክእሉ ጉዳያት ንምፅናዕ ዝተዳለወ መሕትት

001. ኣድራሻ/ ቀበሌ-----------------

002. ቁፅሪ መሕትት-------------------

003. ስም ጥዕና ትካል--------------------------

መዘኻኸሪ**-** ካብቶም ዝተውሃቡ መማረፅታት ሕረ/ዪ፤ ካሊእ ሓሳብ እንተሃልዩ ኣብቲ ክፍቲ ቦታ ይፅሓፉ/ፋ

ክፍሊ ሓደ- ማሕበራውን ኢኮኖሚያውን ኩነታት ኣዴታት

| ተ.ቁ | ሕቶ | መልሲ | | ናብ ዝቕፅል ሕለፍ/ፊ |
| --- | --- | --- | --- | --- |
|  | ናይ ኣዶ ዕድመ ክንደይ ድዩ? | ………….ዓመት | |  |
|  | ኩነታት ሓዳር | 1. ባዓልቲ ሓዳር  2. ዘይተመርዐወት  3. ዝተፋተሐት  4. በዓል ገዝአን ዝሞተን  5. ተፈላልዮም ዝነብሩ  6. ዘይሕጋዊ ሓዳር | |  |
|  | ሃይማኖትክን (እምነትክን) እንታይ እዩ? | 1. ኦርቶዶክስ  2. ሙስሊም  3. ፕሮቲስታንት  4. ካቶሊክ  5. ካልእ ይግለፃ------------- | |  |
|  | ብሄረሰብክን እንታይ እዩ | 1. ትግራይ 2. ኣምሓራ 3. ካልእ ይግለፃ------------- | |  |
|  | ዝነብራሉ ቦታ | 1. ከተማ 2. ገጠር | |  |
|  | ናይ ኣዶ ደረጃ ትምህርቲ | 1. ዘይተምሃረት  2. ቀዳማይ ብርኪ  3. ካልኣይ ብርኪ  4. ኮሌጅን ልዕሊኡን | |  |
|  | ናይ ኣዶ ቀንዲ መተሓዳደሪ ስራሕ | 1.ሙሉእ እዋን ኣብ ገዛ  2. ናይ መንግስቲ ሰራሕተኛ  3. ነጋዴ  4. ናይ ባዕለይ ስራሕ/ትካል ኣለኒ  5. መዓልታዊ ስራሕ  6. ካልእ ይግለፃ------------- | |  |
|  | ወርሐዊ ናይ ገዛ እቶት | -------- ናይ ኢትዮጵያ ቅርሺ | |  |
|  | ናይዚ ሕንጦ ዕድመ | ………. መዓልቲ (ሰዓት) | |  |
|  | ናይዚ ሕንጦ ፆታ | 1.ተባ 2.ኣን | |  |
| ክፍሊ ክልተ- ኩነታት ጥዕና ኣዶ ኣብ እዋን ጥንሲን ሕርስን | | | | |
|  | ክንደይ ቆልዑ ኣለውወን? | | ……………..ብቁፅሪ |  |
|  | ነዚ/ነዛ ህፃን ጥንስቲ እናሃለዋ ቅድመ ወሊድ ክትትል ዶ ገይረን ይፈልጣ? | | 1. እወ  2. ኣይፋለይን | ኣይፋለይን፣  ናብ ተ.ቁ 114 |
|  | ናይ ተ.ቁ 112 መልሲ እወ እንተኮይኑ ክንደይ ግዜ ክትትል ገይረን? | | 1. በዝሒ ክትትል   ………. ብቁፅሪ  2. ኣይዝክሮን |  |
|  | እዚ ህፃን ዝተወለደሉ ቦታ | | 1. ኣብ ገዛ  2. ሆስፒታል  3. ጥዕና ጣብያ  4. ካልእ (ይግለፃ--) --------- | መልሱ ኣብ ገዛ  እንተኮይኑ ናብ  ተ.ቁ 116 |
|  | እዚ ህፃን ዝተወለደሉ ቦታ ኣብ ጥዕና ትካል እንተኮይኑ ዓይነት ወሊድ | | 1. ብንቡር ወሊድ  2. ብመሳርሒ ብምሕጋዝ  3. ብመጥባሕቲ |  |
|  | ዘዋለደን ኣካል | | 1. ናይ ልምዲ መዋለዲት/  2. ጥዕና ፓኬጅ  3. ጥዕና በዓል ሞያ  4.ቤተሰብ (ዘመድ፣ጎረቤት)  5. ካልእ (ይግለፃ) ----------- |  |
|  | እዋን ወሊድ (ሕማም ሕርሲ ካብ ዝጀመረሉ ክሳብ ትወልዳ) | | ………………… ሰዓታት |  |
|  | ዘዋለደን ኣካል ንክንደይ ግዘ ማህፀንኪ ፈቲሹኪ? | | ................ብቑፅሪ |  |
|  | ኣብ እዋን ሕርሲ ረስኒ (ሙቐት) ነይሩክን ድዩ? | | 1. እወ  2. ኣይፋለይን  ግለፂ……… |  |
|  | ናይ ሕርሲ ፈሳሲ ዝተፈለየ (ዝኣጎለ) ሽታ ነይርዎ ድዩ? | | 1. እወ  2. ኣይፋለይን |  |
|  | ነዚ/ዛ ህፃን ጥንስቲ እናሃለክን ናይ ፀቕጢ ደም (ደም ግፊት) ፀገም ኣጋጢሙወን ነይሩ ድዩ? | | 1. እወ  2. ኣይፋለይን  ግለፂ……… |  |
|  | ነዚ/ዛ ህፃን ጥንስቲ እናሃለክን ናይ መድመይቲ ፀገም ኣጋጢሙወን ነይሩ ድዩ? | | 1. እወ  2. ኣይፋለይን |  |
|  | ነዚ/ዛ ህፃን ጥንስቲ እናሃለክን ናይ ሽንቲ ወይ ኩላሊት ረክሲ/ ኢንፌክሽን ኣጋጢሙወን ነይሩ ድዩ? | | 1. እወ  2. ኣይፋለይን |  |
| ንምስታፍክን ኣዚየ የምስግን፡፡ | | | | |
